# Delineating early developmental pathways to ADHD: Setting an international research agenda

**DOI:** 10.1002/jcv2.12144

**Published:** 2023-02-13

**Authors:** Meghan Miller, Anne B. Arnett, Elizabeth Shephard, Tony Charman, Hanna C. Gustafsson, Heather M. Joseph, Sarah Karalunas, Joel T. Nigg, Guilherme V. Polanczyk, Elinor L. Sullivan, Emily J. H. Jones

**Affiliations:** ^1^ Department of Psychiatry & Behavioral Sciences and MIND Institute University of California Davis California USA; ^2^ Division of Developmental Medicine Boston Children's Hospital Boston Massachusetts USA; ^3^ Department of Pediatrics Harvard Medical School Boston Massachusetts USA; ^4^ Department of Psychiatry Faculdade de Medicina FMUSP Universidade de São Paulo Sao Paulo Brasil; ^5^ Institute of Psychiatry, Psychology & Neuroscience (IoPPN) King's College London London UK; ^6^ Department of Psychiatry Oregon Health & Science University Portland Oregon USA; ^7^ Department of Psychiatry University of Pittsburgh Pittsburgh Pennsylvania USA; ^8^ Department of Psychological Sciences Purdue University West Lafayette Indiana USA; ^9^ Centre for Brain and Cognitive Development Birkbeck, University of London London UK

**Keywords:** ADHD, early development, infancy, longitudinal, prevention, prospective

## Abstract

**Background:**

Attention‐deficit/hyperactivity disorder (ADHD) is a prevalent, impairing, and highly heritable condition typically diagnosed in middle childhood. However, it is now recognized that symptoms emerge much earlier in development. Research focused on understanding—using multiple units of analysis—the cascade of early‐life (i.e., prenatal‐infant‐toddler) developmental changes that will later emerge as ADHD has the potential to transform early identification, prevention, and intervention. To this end, we introduce the recently established Early ADHD Consortium, an international network of investigators engaged in prospective, longitudinal studies of risk for ADHD beginning early in life, conducted within a developmental framework, and which incorporate multimethod approaches. This network seeks to harmonize measures and methodological approaches to increase the potential for data sharing and subsequent impact.

**Methods:**

This perspective paper highlights the importance of investigating pre‐diagnostic markers of ADHD, and potential models and mechanisms of ADHD risk and development, with the long‐term objective of facilitating development of preemptive interventions that will minimize the impact of ADHD symptoms on everyday functioning and maximize health and developmental outcomes.

**Results:**

We selectively describe key challenges and questions for this field related to theoretical models and developmental mechanisms in ADHD and recommend next steps for the science, including methodological, measurement, and study design considerations. We then describe potential implications for preemptive intervention development. We conclude by considering other issues including ethical concerns and the critical value of incorporating stakeholder input.

**Conclusions:**

It is hoped that this perspective puts forth a research agenda that will enhance collaborative efforts and accelerate progress in understanding developmental mechanisms and the early ADHD phenotype, with implications for early intervention enhancement of healthy development for infants, young children, and their families.

**Key points**

- ADHD is a prevalent and impairing condition typically diagnosed around age 7, but it is increasingly acknowledged that symptoms emerge much earlier.
- Research focused on understanding the cascade of early‐life developmental changes that occur in ADHD across multiple levels of analysis has the potential to transform early identification and intervention.
- We established the *Early ADHD Consortium*, a network of investigators engaged in prospective, longitudinal studies beginning early in life, conducted within a developmental framework, and which incorporate multimethod approaches; we describe key features of this work in an effort to outline a research agenda for the field.
- We seek to more rapidly advance our understanding of early developmental pathways to ADHD, improving outcomes for infants/young children at risk.

## INTRODUCTION

Attention‐deficit/hyperactivity disorder (ADHD) is an impairing neurodevelopmental disorder with global prevalence estimated at 3%–5% (Polanczyk et al., 2015). Although typically diagnosed in middle childhood (Visser et al., 2014), core ADHD symptoms often emerge in the preschool period and temperamental or other precursors may be apparent even earlier. Affected individuals follow heterogeneous, multifinal developmental pathways and show dysregulation across multiple domains, including attentional control (e.g., maintaining/shifting attention), arousal (e.g., alerting/initiation), executive functioning (e.g., inhibiting a behavior, working memory, planning, organization), and emotion regulation (e.g., regulating internal emotional states and behavioral expressions of emotion) (Faraone et al., 2019). Indeed, ADHD is associated with long‐term impairment (Kuriyan et al., 2013; Larson et al., 2011), often portends other later‐emerging forms of psychopathology (Barbaresi et al., 2013), and frequently co‐occurs with other conditions (e.g., depression [∼15%], anxiety [nearly 20%], oppositional defiant or conduct disorder [∼40%], specific learning disorder [>40%]); the majority of children with ADHD have at least one co‐occurring mental health or neurodevelopmental diagnosis (Cuffe et al., 2020; Larson et al., 2011). Because ADHD is usually the first of these conditions to emerge (Kessler et al., 2014) and appears to exert a causal influence on the later co‐occurring conditions (Riglin et al., 2021; Treur et al., 2021), it plays an outsized role in public health. Its early identification and mechanistic understanding could enable preventive interventions that reduce the incidence of impairing levels of ADHD symptoms but also a range of other impairing, subsequent complications and conditions.

### Why study very early development of ADHD?

There is growing evidence to suggest that the etiological mechanisms associated with ADHD act predominantly before the age at which symptoms can be reliably measured, supporting efforts to investigate developmental precursors and biomarkers associated with ADHD. First, doing so can lead to a more complete understanding of ADHD. ADHD is highly heritable (0.7–0.8; Faraone & Larsson, 2019) and polygenic scores computed from ADHD‐related common genetic variation are at least somewhat predictive of aspects of ADHD (Agnew‐Blais et al., 2021; Groen‐Blokhuis et al., 2014), with neurodevelopmental gene pathways implicated (Cross‐Disorder Group of the Psychiatric Genomics Consortium, 2019). Moreover, established environmental risk factors for ADHD are heavily concentrated in early life including the prenatal and antenatal periods (Kim et al., 2020), although direct causal evidence remains limited. Prenatal risk factors may be related to prenatal stress, poor nutrition, and/or maternal metabolic state as discussed below, and are hypothesized to influence changes in brain development in conjunction with genetic factors which, in turn, contribute to the subsequent emergence of behavioral symptoms.

Second, while not all of these factors are immediate intervention candidates, work in this area has the potential to both (a) identify individuals at risk through more complete early risk indicators, and (b) discover novel ways to intervene to maximize healthy brain development and prevent clinically impairing symptoms of ADHD and other conditions. A key goal is identifying how these factors shape human brain development and behavior, particularly during the fetal, infant, and toddler periods, when experience‐expectant and dependent neural plasticity is maximal, so that novel interventions can be developed that prevent cascading effects of early difficulties and optimize health and functioning. Understanding these early mechanisms may also point to more sensitive and specific markers of ADHD risk that will facilitate early identification more broadly, a topic we turn to later.

### What do we know?

Existing knowledge of early manifestations and precursors of ADHD comes from several sources including retrospective studies and chart reviews, large community‐based samples not selected for ADHD risk (Arnett et al., 2013; Johnson et al., 2014; Willoughby et al., 2017), prospective studies of other conditions (e.g., autism, preterm infants) (Miller et al., 2018), and, more recently, prospective studies of infants at familial risk for ADHD (Auerbach et al., 2008; Miller et al., 2020; Reetzke et al., 2022; Sullivan et al., 2015). Combined, this work suggests that broad and non‐specific phenotypic differences among infants and young children who later develop ADHD may be evident as early as the first year of life. Recent reviews (Johnson et al., 2015; Nigg et al., 2020; Shephard et al., 2022) highlight the range of early developmental features that have been associated with later ADHD, including decreased rate of head growth and cortical volume in the first year, reduced visual‐motor integration, atypical achievement of motor and language milestones, and early temperamental features (e.g., increased irritability, low adaptability, increased negative affectivity). Many of these factors are non‐specific, linked to other neurodevelopmental outcomes (e.g., autism; Johnson et al., 2015) and risk factors (e.g., preterm birth; Cassiano et al., 2020). With respect to core ADHD symptom dimensions in early development, the literature is mixed. Increased activity level among toddlers at familial risk for ADHD or autism who develop elevated ADHD symptoms (but not autism) by preschool has been reported in some samples (Miller et al., 2020; Reetzke et al., 2022) but this is not as clear among infants (Johnson et al., 2014). In one sample, atypical development of sustained visual attention from 3 to 24 months was found among infants at familial risk for autism who later developed ADHD (in the absence of autism) (Miller et al., 2018). Current challenges include that effect sizes for individual predictors remain, on average, relatively small or non‐specific and poorly understood at a mechanistic level, and that there is a lack of research examining the dynamic interplay among predictors (Tobarra‐Sanchez et al., 2022). Second, full clinical syndromes are not apparent in the infant and toddler periods; instead, investigators must rely on precursive measures such as temperament, early cognition, measures of self‐regulation, and others that are only partially related to actual clinical features later. Identifying the developmental linkages here is a key goal (Nigg et al., 2020). Third, harmonizing findings from selected versus population samples, and prospective and retrospective designs, remains needed.

Investigations of the early development of other neurodevelopmental conditions like autism have provided insights (Szatmari et al., 2016) that may inform research on the developmental unfolding of ADHD early in life, although modified ascertainment designs may be needed. For example, prospective studies focused on infant siblings of children with autism have affirmed that the earliest signs of a clinical condition do not necessarily share the same surface features as its later behavioral manifestation (Johnson et al., 2015; Szatmari et al., 2016; Talbott & Miller, 2020). Early precursors of future ADHD may therefore appear across a broader range of phenotypes such as negative affect, differing attentional patterns across ages, and to‐be‐identified metabolic, biological, cognitive, neural, and/or behavioral features. Prospective autism infant sibling studies have also, however, supported the contention that neurodevelopmental differences emerge prior to overt symptoms, supporting the inclusion of neural and metabolic as well as behavioral features in these studies. Unsurprisingly, this work suggests that correlated features, such as infant negative affectivity, are likely to be non‐specific indices of risk, exemplifying both equifinality and multifinality (Cicchetti & Rogosch, 1996). Nonetheless, these can be extremely useful when paired with other features in prospective designs. In fact, these prospective infant sibling studies are ideally‐suited to examine equifinal and multifinal outcomes given the documented higher likelihood of ADHD diagnosis (in the absence of autism) among younger siblings of children with autism or ADHD compared to younger siblings of children with no known diagnosis (though, importantly, the majority of younger siblings do not develop either condition) (Miller et al., 2019a). Thus, the focus on a single outcome (e.g., ADHD diagnosis), or a presumption of specificity, may obscure these patterns and lead to inaccurate causal models. Finally, these prospective studies emphasize that trajectories of change and/or stability (vs. transiency) of concerns may be more robustly predictive of outcomes than data obtained at a single timepoint, signaling the value of repeated measurements over time which allow trajectory modeling.

### Setting a research agenda

Increasingly sophisticated theoretical frameworks for understanding the emergence of neurodevelopmental disorders across genes, brain, and behavior are emerging but have yet to be fully integrated into most empirical work. Current approaches mainly support identification of ADHD once children experience significant impairment, typically not until early or middle childhood. Specification of theoretical frameworks for ADHD with a focus on precursors in infancy and early childhood could guide the field more effectively, increasing the efficiency of targeted hypothesis testing within high‐resource studies and the prospects for the development of earlier interventions. Here, we highlight the importance of investigating pre‐diagnostic markers of ADHD with the long‐term objectives of (1) better understanding causal mechanisms, developmental pathways, and predictive markers; (2) facilitating early identification; and (3) supporting the development of preemptive interventions that will shift trajectories to enable maximal health and development. We begin by briefly reviewing relevant theoretical models and mechanisms. We then describe promising methods, measurement strategies, and study designs with which to undertake this work. Next, we highlight the ways in which research focused on developmental mechanisms and the early ADHD phenotype could be used to inform the development of preemptive interventions. We conclude by considering ethical issues, as well as the critical role of stakeholder input. Throughout, we emphasize the importance of cross‐site collaborative work through research networks focused on these goals, including the newly‐formed *Early ADHD Consortium*.

## CAUSAL MECHANISMS AND PREDICTIVE MARKERS

### Models and mechanisms

Characterization of developmental features that presage later ADHD is critical to building and refining robust theoretical models. Because neurocognitive changes observed in children with ADHD could be secondary to experiential and environmental alterations evoked by early‐emerging symptoms, prospective studies are essential to identifying temporal precedence, a key component of causal models. Ideally, such studies would begin in pregnancy. Many etiological risk factors for ADHD are shared with other neurodevelopmental conditions, and transdiagnostic mechanisms, such as inflammation, have been suggested (Dunn et al., 2019; Graham et al., 2022; Gustafsson et al., 2020). The fetal programming hypothesis (Monk et al., 2019; Wadhwa et al., 2009) proposes that alterations in the fetal environment may lead to adaptive changes in the fetus that, if later mismatched with the postnatal environment, can cause psychopathology; the nature of these mismatches may be one route through which diagnostic specificity occurs.

Prenatal risk factors are often associated with genetic risk (that is, passive genotype‐environment correlation), complicating distinctions between genetic and environmental effects. For example, well‐designed adoption and sibling cohort studies have recently challenged the presumed causal role of maternal smoking on ADHD (Thapar et al., 2009) while affirming other effects (e.g., pregnancy stress, obesity, low birth weight; Kim et al., 2020). In other neurodevelopmental conditions like autism, a graded liability model has been demonstrated, whereby the effect of rare and common variants, sex, and likely environmental factors act additively to increase risk (Antaki et al., 2022). Diagnostic specificity may be driven by common variant profiles shaping complex brain systems, whereas broader vulnerability may result from rare, more penetrant mutations and large environmental impacts (Myers et al., 2020; Niemi et al., 2018). Indeed, there is some evidence that copy number variations and single gene disrupting mutations, of the kind associated only with autism and/or intellectual disability, are also associated with the ADHD phenotype (Harich et al., 2020; Thapar, 2018). Moreover, with the publication of the first genome wide association study to identify allelic variants of significance for ADHD in 2019 (Demontis et al., 2019), the potential to investigate polygenic risk and neurogenetic pathways, along with their interactions with environmental factors (Havdahl et al., 2022), as causal mechanisms for ADHD has been enhanced.

Genetic and environmental risk factors likely exert their influence in large part by altering neurocognitive development. Although cognitive correlates of ADHD in childhood are well described (Willcutt et al., 2005), most of the work conducted in infancy has focused on broad indicators (e.g., overall development) or has used questionnaire or behaviorally‐coded indices (Shephard et al., 2022) that may be only modestly correlated with experimental measures of neurocognitive function. Although these subjective measures can be useful for clinical prediction, other methods are required to dissect precise cognitive mechanisms. One approach is to assume continuity in the cognitive features altered across development; this would favor identifying paradigms that reliably challenge control of top‐down attention but do not require complex expressive or receptive language skills or motor responses. However, many cognitive functions associated with childhood ADHD (working memory, response inhibition, arousal regulation) exist in only vestigial form in infants and toddlers, and some measures, such as attentional look times in infants, require careful interpretation that may not be isomorphic with childhood measures of related constructs. Models that address these challenges deserve heavier use in studies of the emergence of psychopathology and are a key target for this consortium.

Other models highlight early temperamental differences such as low effortful control (i.e., self‐regulation) and high surgency (i.e., extraversion/positive affect) and negative affect as risk markers for ADHD. Several small studies have found that high negative affect and low attentional control are related to familial ADHD risk as early as 6 months of age (Auerbach et al., 2008; Sullivan et al., 2015). Recent results from large‐scale, population‐based samples not selected for ADHD risk have documented elevated temperament‐based activity levels in the first 30 months of life in relation to later ADHD diagnosis or symptoms (Tobarra‐Sanchez et al., 2022) and ADHD polygenic risk scores (Riglin et al., 2022). Other large studies have amplified these findings in relation to developmental cascades by highlighting the moderating role of early caregiving (Miller et al., 2019b). Distinct temperamental profiles may lead to an equifinal ADHD outcome (Kerner auch Koerner et al., 2018) but with partially independent associations to symptom domains and co‐existing psychopathology (Bunford et al., 2021; Martel et al., 2009; Rutter & Arnett, 2021). In addition, understanding links between temperamental variation and early trajectories of cognitive and brain function is important, given the emerging hypothesis that excessive early infant negative affect may causally disrupt development of top‐down cognitive control in the preschool years (Nigg et al., 2020; Rabinovitz et al., 2016). Thus, a major goal of studies organized by the consortium is to clarify patterns of early temperament development and the transactional influences of early affect and attention development on each other.

Direct measures of brain function may be highly translatable over development and across species, allowing greater traction for identifying biological mechanisms. For example, there has been an explosion of interest in aperiodic activity in the electroencephalogram (EEG) power spectrum, which is theorized to reflect cortical excitation/inhibition balance (Ostlund et al., 2022) and appears related to both ADHD familial risk in infancy and ADHD diagnosis in later childhood in cross‐sectional samples (Karalunas et al., 2022). Interestingly, these effects may also be shared with autism (Shuffrey et al., 2022). The most investigated EEG feature in ADHD is the enhanced theta‐beta ratio, which is a measure of slow relative to fast neural oscillatory power and reflects both periodic and aperiodic influences. One recent study identified alterations in this ratio among infants with familial ADHD risk (Begum‐Ali et al., 2022). However, theta‐beta ratio effect sizes in diagnosed children have decreased over time (Arns et al., 2013), possibly due to clinical and etiological heterogeneity in ADHD, as well as inclusion of more representative comparison samples in recent research (Alperin et al., 2019). Other studies in infants have focused on single bands (e.g., theta) to differentiate clinical risk (Shephard et al., 2019). An important direction for the field (and this consortium) is clarifying which alterations in brain function are most sensitive and specific to ADHD risk.

Family environment is likely a cross‐cutting moderator of early brain‐ and behaviorally‐based developmental trajectories associated with ADHD. However, evidence linking specific familial characteristics to ADHD outcomes remains sparse. Relevant dimensions include stress and parenting quality/styles (e.g., sensitivity/scaffolding during infancy/early childhood, discipline, monitoring, authoritarian/authoritative style at older ages), parental relationship status, and parental maltreatment, among others (Claussen et al., 2022; Miller et al., 2019b). The impact of parental ADHD on the home environment may be highly nuanced and can include increased chaos and reduced socioeconomic resources, greater parental empathy, and/or increased dyadic emotional conflict escalation. Reciprocal‐transactional processes among child‐level and contextual factors (e.g., parent behavior) are likely significant (Agnew‐Blais et al., 2022). Mitigating interventions may prevent cascading effects while enhancing compensatory factors, but the role of gene‐environment correlations must be considered. A major goal of the *Early ADHD Consortium* is to undertake studies that span levels of analysis to clarify how the early family environment affects neural, cognitive, behavioral, and emotional development to either potentiate or mitigate risk.

### Methods, measurement, & study design

Research on early precursors to ADHD has taken many forms, but the use of familial risk designs in this population is growing. Such designs rely on recruitment of infants who have a family history of ADHD, which has typically been defined as having a diagnosed first‐degree relative (i.e., older sibling or parent). Some studies have focused on pregnant mothers with ADHD, following their offspring over time (Gustafsson et al., 2020; Sullivan et al., 2015). As described earlier, these designs have produced novel and intriguing findings in recent years (Begum‐Ali et al., 2022; Joseph et al., 2022; Miller et al., 2021; Sullivan et al., 2015), suggesting that they are both feasible and fruitful.

Research focused on infants with elevated likelihood for ADHD has traditionally relied heavily on parent or observer reported temperament and behavior. Many early behavioral rating tools are psychometrically strong but lack normative samples that would support their translation into clinical decision‐making. Similarly, while core domains affected by ADHD are thought to be relevant across the lifespan, their behavioral expression varies widely across development (i.e., heterotypic continuity), limiting measurement continuity in longitudinal research. Moreover, the validity of measuring *DSM*‐defined ADHD symptoms in toddlerhood is complicated by the fact that many of these behaviors are considered developmentally appropriate at this age.

Although some ADHD rating scales are validated for use as early as 24 months of age, the majority of reliable measures are normed for preschool‐age children or older (øvergaard et al., 2018; Rimvall et al., 2014). Parent/caregiver report should be a component of any study focused on the early development of ADHD as they provide a unique perspective on the infant/young child within the home and family environment. That said, over‐reliance on parent/caregiver report presents challenges regarding differences in caregiver understanding of normative development and behavior and the potential influence of parental symptoms and/or having an older child with ADHD to bias the caregiver's perspective and ratings. Direct observation, especially observations made by coders unaware of the child's family history or prior evaluations, is a valuable addition to studies focused on the early ADHD phenotype, but can be labor‐intensive. Beyond child‐specific symptoms/behavior, other potentially useful measurement tools include parent‐child interaction activities; standardized developmental measures; and measures of contextual factors such as family environment, parenting stress, and neighborhood factors.

Methods without behavioral demands are useful for testing many of the models described in the previous section including eye‐tracking, pupillometry, heart rate, and accelerometry (wearable motion‐based activity monitors). These methods are feasible for repeated administration across early development but have been used less frequently despite theoretical support (Mehta et al., 2019; Unsworth & Robison, 2017; Weigard et al., 2018) and well‐defined links to central and autonomic nervous system functioning, cognitive performance, and self‐regulation. Such objective approaches have the potential to reveal mechanisms and identify early differences that distinguish infants who go on to develop ADHD from those who do not. Noninvasive brain imaging methods (EEG, near infrared spectroscopy, MRI) are also increasingly being used with infants and toddlers (Goodwin et al., 2021a; Karalunas et al., 2022), and novel fetal brain imaging methods are emerging (Thomason, 2020). These methods may provide increased objectivity, but questions remain about clinical utility, scalability, and translational potential. Machine learning approaches based on raw data inputs are increasingly being used for biomarker identification (Das & Khanna, 2021) and predictive modeling (Senior et al., 2021), though less often prospectively from infancy. A major challenge for the field is how to combine data‐driven approaches (i.e., atheoretical use of all possible input features from raw/minimally processed data that allow researchers to explore a broad spectrum of potential predictive biomarkers but can also produce difficult‐to‐interpret results with minimal mechanistic relevance) with theory‐driven models (i.e., using a smaller set of theoretically‐selected features from more highly‐processed data, combining features via factor analysis) that may miss yet‐to‐be‐discovered associations but yield more interpretable insight into putative developmental mechanisms.

The translational utility of pre‐diagnostic markers of childhood ADHD is likely to improve with longitudinal measurement. Indeed, evaluation of the models described in the previous section requires prospective collection of objective measurements that can be consistently administered over development. Developmental trajectories of key measures may offer increased predictive power over statistical differences in single‐timepoint measures, and present opportunities to model gene‐neurobiology‐environment transactions. Logistical challenges unique to longitudinal work include high costs, need for funding renewals to follow precious infant samples to ages at which diagnostic outcomes can be obtained, participant retention, and labor‐intensive data collection procedures. Moreover, since only a subset of infants will develop the full ADHD phenotype, increasingly large samples are needed.

Another key issue facing this work relates to defining outcomes. Often, prospective studies work backward from diagnostic outcomes to determine which earlier measures may distinguish those who develop the disorder from those who do not across domains and time. Such approaches have high clinical utility, as eventual clinical decision‐making requires categorical stratification and cutoffs, but may be less equipped to reveal mechanisms involved in symptom development, particularly not in a transdiagnostic manner. Indeed, there is a growing interest in using dimensional frameworks to understand psychopathology (e.g., RDoC) since dimensions are better suited to understanding associations between variables, and better capture the full range of phenotypic differences among those who do and do not meet diagnostic thresholds for specific neurodevelopmental conditions (i.e., diagnostic‐agnostic approaches) (Cuthbert, 2014). Dimensional approaches are particularly well‐suited to investigate high‐risk samples such as those comprised of infants at familial risk for ADHD, which are enriched with a wide range of phenotypic variation. Therefore, studies may do well to balance and integrate categorical (i.e., ADHD vs. not) and dimensional (i.e., continuous symptoms, level of impairment) approaches to outcome measurement; prospective “infant‐first” analytic techniques like latent class analysis may also yield new insights into the latent structure of neurodevelopmental conditions.

Across methods, it will be critical to establish developmental, age‐based norms for measures that emerge as candidate predictors or biomarkers and to have the potential for clinically actionable cut points to be identified. This will increase the likelihood that such measurements could be used clinically and will require development of large‐scale databases via pooled studies with overlapping designs. We anticipate that assessment ages and measurement tools will vary across studies, introducing statistical complications. Use of continuous age in planned analyses will allow for easier pooling across multiple cohorts; likewise, data harmonization efforts may be possible when there is overlap in constructs measured. Recent examples in the child mental health literature have provided a model for such harmonization processes at a national level, involving contributions from researchers, clinicians, and community agencies (Boulton et al., 2021). Such models could be scaled internationally and targeted toward infancy/early childhood; our consortium is a step toward that goal. Although we do not intend to prescribe a standard list of measures here, we wish to emphasize that collaboration, among this group and with others, is the first step toward developing such a list; we provide an overview of potential measures in Table 1.

**TABLE 1:** Assessment and measurement tools utilized by the early ADHD consortium investigators.

Domain	Direct child assessment	Caregiver report
Adaptive functioning		Adaptive behavior assessment system^a^ Vineland adaptive behavior scales^a^ Bayley social emotional and adaptive behavior questionnaire^b^
ADHD	Behavior rating inventory for children (BRIC)^b^ DSM‐5 ADHD checklists^c^	Child behavior checklist 1.5–5^b^ ^,^ ^f^ ADHD rating scale, preschool^c^ Kiddie schedule for affective disorders and schizophrenia (K‐SADS)^c^
Attention/Executive functioning	Eye‐Tracking^a^ Visual attention task^a^ Delay Aversion/Snack Delay^b^ Flanker task^c^ Go‐No‐Go^c^ NEPSY‐II statue test^c^ Simon task^c^ Unicorn/Dragon or day/Night tasks^c^	
Family and medical history		Child demographics and perinatal history Maternal nutrition O'Leary‐Porter scale Parental drug use Perinatal substance use^a^ Infant/Toddler health and feeding^a^ Intervention, medical and developmental history^a^
Family psychiatric and ADHD traits		Adult ADHD rating scales^d^/Adult ADHD self report scale^d^ ^,^ ^f^ Alcohol, smoking, and substance involvement screening test^d^ Anxiety and depression inventories, including during perinatal/postnatal periods^d^ Diagnostic interview for ADHD in adults^d^ ^,^ ^f^ Structured clinical interview for DSM‐5^d^ Conners 3^rd^ Ed.^e^ Child & adolescent symptom inventory 5^th^ Ed. (parent, teacher)^e^ K‐SADS^e^
Global development	Infant behavior record^a^ Mullen scales of early learning^a^ Bayley scales of infant Development‐3^a^ Developmental profile^a^ Wechsler preschool & primary scale of Intelligence‐IV^b^ Bracken school readiness assessment^c^	Ages and stages questionnaire^a^
Language/Communication		MacArthur‐Bates communicative development inventory^a^
Other behavior	Play observation^a^ Disruptive behavior diagnostic observation schedule^c^	Multidimensional assessment of preschool disruptive behavior^b^ Disruptive behavior disorders rating scale^c^ Kiddie‐disruptive behavior disorders schedule^c^
Parenting/Environment	Home observation measurement of the environment^a^ Parent‐child interaction^a^	Maternal antenatal and postnatal attachment scale^a^ Social support inventory^g^ Confusion, Hubbub, and order scale^a^ Co‐parenting relationship scale^a^ Parenting daily hassles^a^ Parenting sense of competence scale^a^ Parenting stress index^a^
Psychopathology		Behavior assessment system for Children‐3^b^ Brief infant‐toddler social and emotional assessment^b^ Child behavior checklist 1.5–5^b^ Children's behavior questionnaire^c^ Strengths and difficulties questionnaire^c^ Preschool age psychiatric assessment^b^
Sensory‐motor	Accelerometry^a^ Sensory processing assessment^b^	Infant/Toddler sensory profile^a^
Sleep	Actigraphy^a^	Brief infant sleep questionnaire^a^ Brief toddler sleep inventory^c^ Children's sleep habits questionnaire^c^
Temperament and self‐regulation	Devereux early childhood assessment for infants and toddlers^a^ Still face paradigm^a^ Strange situation^a^ Lab‐TAB^a^	Infant behavior questionnaire^a^ Early childhood behavior questionnaire^b^
Vital signs and neurobiology	Cortisol^a^ Heart rate variability^a^ Inflammatory markers^a^ NICU network neurobehavioral scale^a^ Resting EEG^a^ Passive ERP^b^	

*Note*: Youngest age of administration.

^a^ <1 year.

^b^ 1–2.9 years.

^c^ ≥ 3 years. Proband status.

^d^ Parent.

^e^ Sibling.

^f^ Available in languages other than English.

^g^ Prenatal.

### Recommendations

Taken together, research on early mechanisms and predictive markers involved in the development of ADHD must involve multidimensional, longitudinal assessment in samples large enough to sufficiently model these reciprocal interactions among genes, environment, neurocognitive function, and behavior. This will involve multi‐site, international collaborations reliant on shared data. The potential for sensitive neurodevelopmental periods must be considered because this may inform intervention timing. Further, developmental cascade models suggest that events occurring at a particular point in development affect later milestones (Oakes & Rakison, 2019). Questions remain regarding the extent to which temperament traits influence development of core ADHD symptoms, and the extent to which these traits are modifiable. Further considerations include specificity of putative (or identified) mechanisms, and conversely the benefits of transdiagnostic and dimensional approaches to understanding the early emergence of ADHD and its constituent symptoms (Pacheco et al., 2022). A focus on transdiagnostic, or cross‐cutting, early risk factors (e.g., temperament‐related differences) may provide both theoretical benefits and be more pragmatic for clinical systems (Talbott & Miller, 2020).

**FIGURE 1:**
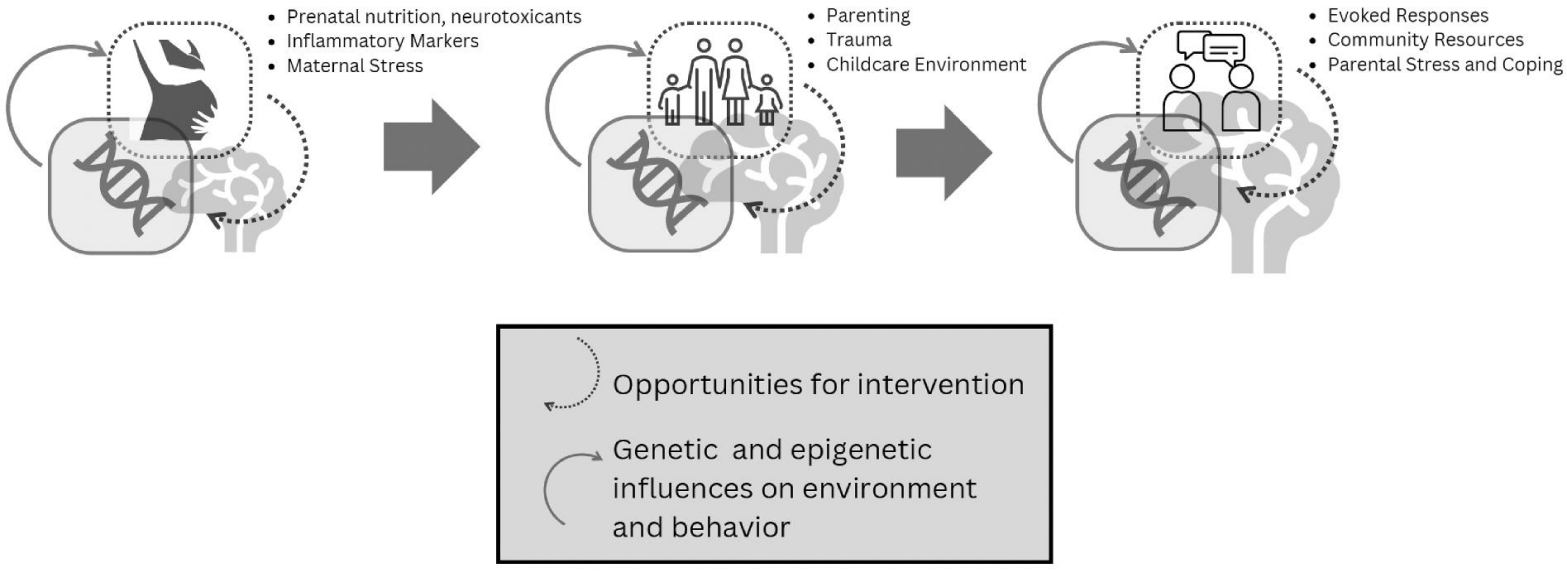
Schematic depiction of the cascade model of neurodevelopment in which bidirectional influences between genetic and environmental factors impact brain and behavioral development. Dashed arrows indicate effects of environment on genetic expression and brain/behavior outcomes, which are potential opportunities for intervention. Solid arrows indicate effects of genetic and epigenetic expression, which may be harder to moderate. *Early ADHD Consortium* members are actively probing these pathways.

As such, we propose that multimethod, multi‐informant approaches to the study of the early ADHD phenotype are likely to be most informative. As illustrated in Figure 1, these should encompass dense longitudinal multimodal measurements to capture unfolding gene‐brain‐body‐environment interactions. The resulting data should be analyzed with both sophisticated data‐driven and theoretically‐informed approaches, coupled with appropriate use of open science tools like pre‐registration. To move closer to causal inference, the field should incorporate tools like propensity‐score matching and directed acyclic graphs, genetically‐informed designs, and embedded shiftability or experimental intervention approaches. Finally, a focus on replication and generalization within a regulatory framework is important for yielding clinically utilizable biomarkers (Loth & Evans, 2019).

## DEVELOPMENT OF PREEMPTIVE INTERVENTIONS

Another key consideration in our efforts to identify early developmental pathways to ADHD is whether and how we may intervene on any identified predictive processes. In‐depth investigation of early‐life precursors to ADHD and their developmental trajectories is crucially needed in this regard, as identifying changes in early behavior or development may reveal ideal timepoints and targets for intervention. Currently, there is no clear way to identify which infants/young children would benefit from interventions. Thus, programs targeting broad domains of development with hypothetical benefits for the general population could be of interest as testable preemptive interventions for ADHD. Candidate programs include those focused on improving broad aspects of pre‐ and perinatal care (e.g., home‐visiting programs; Olds et al., 2014), as well as early childhood programs (e.g., Head Start; Bauer & Whitmore Schanzenbach, 2016) and preschool interventions targeting self‐control/executive functions (Halperin et al., 2020).

Downward extensions of traditional parent‐training programs that promote positive parenting and support the development of children's emotional and behavioral regulation are also potential candidates (e.g., New Forest Parenting Program; Sonuga‐Barke et al., 2018), Incredible Years (Beauchaine et al., 2015), Positive Parenting Practice/Triple P (Sanders et al., 2014). A meta‐analysis of RCTs evaluating the efficacy of these or similar early interventions for ADHD in the toddler and preschool years reported significant improvements in ADHD symptoms when measured immediately post‐intervention with a moderate standardized mean difference effect size of 0.43 across all types of intervention (Shephard et al., 2022), but long‐term efficacy is unclear and there was little evidence of improvement in other outcome measures (e.g., executive function processes). Approaches that incorporate treatment for parents with ADHD are also promising (Chronis‐Tuscano et al., 2017) and may address issues related to the deleterious effects of caregiver ADHD on parenting practices (Oliveira et al., 2022) as well as adherence to, and efficacy of, parent‐mediated interventions for infants and very young children at risk for ADHD.

Alternatively, studies may target specific developmental processes that are hypothesized to underlie ADHD emergence using technology‐based methods that are less costly and burdensome to parents. The only randomized controlled trial to test the efficacy of an attentional control training program in infants with familial risk for ADHD (aged 9–16 months) reported no immediate treatment effect on attentional and inhibitory control processes, or on other early alterations associated with ADHD (e.g., elevated activity levels; Goodwin et al., 2021b). However, it remains possible that effects may not be fully revealed until later in development.

Future research on early or preemptive interventions for ADHD will benefit from a better understanding of early life mechanisms and precursors, which may inform our development of more targeted treatments and our understanding of “active ingredients.” Questions about whether such interventions could change trajectories of risk if applied early enough in life will require well‐powered, longitudinal, randomized controlled trials. In addition, changes in broad‐based functional outcomes (e.g., academic, social) are under‐explored but could be arguably more important than core ADHD symptom reduction in some cases. However, the large sample sizes required to demonstrate effects (particularly the absence of emergence of impairments), along with longer follow‐up periods required to reveal potential latent effects, remain challenges.

## ETHICAL ISSUES AND COMMUNITY ENGAGEMENT

Several ethical issues arise related to implications of research focused on early identification of, and intervention for, ADHD/ADHD risk, including concerns about potential risks of false positives. Although research on preschoolers with ADHD has demonstrated relatively high stability of preschool diagnoses in clinically‐referred samples (without consideration for subtype/presentation) into middle childhood (Lahey, 2004; Riddle et al., 2013), stability of even earlier diagnoses remains unclear at best. Stability of infant or toddler risk factors and strength of associations with later psychopathology is less certain still and likely to have some limitations. Thus, the potential for low stability of very early precursors or even preschool diagnoses in non‐referred samples could result in increased familial stress, stigma, and iatrogenic effects on children and families, along with increased stress to care systems related to unnecessary interventions. Insurance denials or other inappropriate intrusions into privacy are another risk of any screening program. Another challenge relates to lack of availability of adequate evidence‐based resources and/or preemptive interventions for families of very young children with or at risk for ADHD—hence the need for strong, mechanistic discovery as highlighted above. These considerations must be balanced carefully against potential broad‐based benefits of earlier intervention for ADHD and prevention of serious later outcomes.

Another ethical consideration relates to medication, one of the two evidence‐based treatments for ADHD, but not for mere risk factors or precursive signs or for infants or toddlers. Current guidance (e.g., American Academy of Pediatrics, UK NICE Guidelines) specifies behavioral interventions as the recommended first‐line treatment for ADHD in children under the age of 6. Concerns could be raised about whether earlier identification of very young children at markedly elevated risk for ADHD might lead to inappropriate prescribing of medication in very young children (Burcu et al., 2016; Raman et al., 2018). However, earlier identification of key precursors or of ADHD and its constituent symptoms could, perhaps more promisingly, provide opportunities for the eventual development of many kinds of safe, effective development‐promoting interventions, from pregnancy‐related dietary, supplemental, or health interventions to behaviorally‐based, early life caregiver‐implemented interventions that could reduce the need for later intensive support/treatment and maximize the child's longer‐term healthy development. For example, folate supplementation in pregnancy is routine to prevent spina bifida, Vitamin C supplementation can reduce the respiratory effects of maternal smoking in pregnancy on infants, and preliminary evidence suggests that sufficient omega‐3 intake may rescue the adverse effects on offspring temperament of maternal unhealthy diet (Gustafsson et al., 2019). Thus, the leverage provided by early life intervention may provide new ways to think about powerful intervention for maximal health and development in relation to psychopathology.

Finally, and importantly, there are concerns in the broader neurodiversity community regarding the ethics of preemptive interventions. Ensuring research is aligned with the priorities of the ADHD and parent communities is paramount to building trust, uncovering new research directions, and maximizing the impact of ADHD research. Input from people with lived experience of ADHD can provide critical insights into challenges and strengths (Sedgwick et al., 2019) that may be important topics for further research and may identify new targets for strengths‐based early interventions. Goals of any potential preemptive interventions should be community‐informed and would likely focus on preventing the challenges/impairment associated with ADHD and the identification of resilience factors. This could involve not only research at the level of the individual and family, but also a focus on structural changes that may allow more children to experience a match between their skills and environments. Involvement of various stakeholders in discussions about these issues through community‐based participatory research approaches will be important to developing a robust understanding of an acceptable clinical research agenda to the ADHD community and will help researchers ensure this work is less likely to be framed in ways that perpetuate stigma. In particular, while there is recognition of the need to involve ADHD community members as stakeholders, there is relatively little work using formally‐defined methods (but see Adamou et al., 2016) such as participatory action research methods (Shamrova & Cummings, 2017) or cultural consensus modeling (Ulijaszek, 2013) that would allow individuals with ADHD and their family members to actively participate in defining research questions, planning and conducting studies, and interpreting and disseminating findings. Ultimately, novel early identification and intervention approaches will only be widely adopted if they are welcomed by individuals with ADHD and their families. Our consortium, which includes members with dual roles (e.g., researcher and family member), hopes to pursue this.

Community engagement is also necessary to identifying strategies to study early ADHD in historically underserved groups within which differences in prevalence or presentation are observed, including people from lower resourced backgrounds (Russell et al., 2016), women and girls (Hinshaw et al., 2022), and minoritized groups based on race and/or ethnicity. Issues of distrust in research institutions abound, necessitating a change in the dialogue surrounding what research aims to accomplish via clearly communicated scientific goals that are community‐informed. It is likewise important to engage the clinical community that serves individuals with ADHD in delineating an appropriate clinical research agenda to ensure that any resulting tools (e.g., early screening or diagnostic measures) or intervention approaches will be acceptable to those providing services.

## CONCLUSIONS

ADHD is a prevalent and impairing condition that tends to be diagnosed in middle childhood despite growing agreement that earlier behavioral and biological differences are detectable. Understanding the cascade of early‐life changes that occur in response to proximal genetic and environmental risk factors for ADHD has the potential to transform early identification and intervention, as such mechanisms shape brain development and behavior. Identifying infants and young children on a path to ADHD may allow for the development and implementation of earlier or preemptive interventions that prevent compounding effects of early difficulties and optimize outcomes. Work in this area will require prospective, longitudinal studies of infants with a family history of ADHD beginning early in life (or prenatally), conducted within a developmental framework, and which incorporate multimethod approaches. These studies will benefit from multi‐site, international collaborations and networks that may share common methods and objective measures and which incorporate stakeholder perspectives, such as our recently‐established *Early ADHD Consortium*. The ultimate goal is to more rapidly advance our understanding of early developmental pathways to ADHD that will improve outcomes for infants and young children.

## AUTHOR CONTRIBUTIONS

**Meghan Miller**: Conceptualization; Project administration; Supervision; Writing – original draft; Writing – review & editing. **Anne B. Arnett**: Conceptualization; Writing – original draft; Writing – review & editing. **Elizabeth Shephard**: Conceptualization; Writing – original draft; Writing – review & editing. **Tony Charman**: Conceptualization; Writing – review & editing. **Hanna C. Gustafsson**: Conceptualization; Writing – original draft; Writing – review & editing. **Heather M. Joseph**: Conceptualization; Writing – original draft; Writing – review & editing. **Sarah Karalunas**: Conceptualization; Writing – original draft; Writing – review & editing. **Joel T. Nigg**: Conceptualization; Writing – review & editing. **Guilherme V. Polanczyk**: Conceptualization; Writing – original draft; Writing – review & editing. **Elinor L. Sullivan**: Conceptualization; Writing – original draft; Writing – review & editing. **Emily J.H. Jones**: Conceptualization; Writing – original draft; Writing – review & editing.

## CONFLICTS OF INTEREST STATEMENT

Dr. Charman has served as a paid consultant to F. Hoffmann‐La Roche Ltd. and Servier; and has received royalties from Sage Publications and Guilford Publications. Dr. Polanczyk has served as a consultant/speaker to Abbott, Ache, Medice, Novo Nordisk, and Takeda, and has received royalties from Editora Manole. He is also a Joint Editor for JCPP *Advances*. Dr. Nigg has received royalties from Guilford Publications, he also serves on the JCPP *Advances* Editorial Advisory Board. Elizabeth Shephard serves on the JCPP *Advances* Editorial Advisory Board. Emily J.H. Jones is a Joint Editor for JCPP *Advances*. All other authors have declared that they have no competing or potential conflicts of interest.

## ETHICAL CONSIDERATIONS

No ethics approvals were required for the preparation of this research review, as no patients or members of the public were involved.

## Data Availability

Data sharing not applicable to this article as no datasets were generated or analyzed.
